# Conformer-Based Human Activity Recognition Using Inertial Measurement Units

**DOI:** 10.3390/s23177357

**Published:** 2023-08-23

**Authors:** Sowmiya Seenath, Menaka Dharmaraj

**Affiliations:** Noorul Islam Centre for Higher Education, Kanyakumari 629180, TamilNadu, India; menakaberita@gmail.com

**Keywords:** conformer, transformer, human activity recognition, inertial measurement units, wearables

## Abstract

Human activity recognition (HAR) using inertial measurement units (IMUs) is gaining popularity due to its ease of use, accurate and reliable measurements of motion and orientation, and its suitability for real-time IoT applications such as healthcare monitoring, sports and fitness tracking, video surveillance and security, smart homes and assistive technologies, human–computer interaction, workplace safety, and rehabilitation and physical therapy. IMUs are widely used as they provide precise and consistent measurements of motion and orientation, making them an ideal choice for HAR. This paper proposes a Conformer-based HAR model that employs attention mechanisms to better capture the temporal dynamics of human movement and improve the recognition accuracy. The proposed model consists of convolutional layers, multiple Conformer blocks with self-attention and residual connections, and classification layers. Experimental results show that the proposed model outperforms existing models such as CNN, LSTM, and GRU. The attention mechanisms in the Conformer blocks have residual connections, which can prevent vanishing gradients and improve convergence. The model was evaluated using two publicly available datasets, WISDM and USCHAD, and achieved accuracy of 98.1% and 96%, respectively. These results suggest that Conformer-based models can offer a promising approach for HAR using IMU.

## 1. Introduction

The physical activities that a person engages in can offer valuable information about his/her daily routine, health conditions, habits, and even mental state. Monitoring the timing and manner in which individuals carry out physical activities can yield valuable data for the customization of fitness routines, detection of possible health problems, and promotion of overall well-being. There are many different fields in which human activity recognition (HAR) can be applied due to its diverse range of potential applications. Some of the prominent areas are healthcare monitoring, sports and fitness tracking, video surveillance and security, smart homes and assistive technologies, human–computer interaction, workplace safety, and rehabilitation and physical therapy.

Most of the earlier works on activity recognition system rely upon information from videos and images. The increased demand for automated systems has augmented the complexities of the systems, and challenges associated with image data have also increased. While considering an image or video, various factors should be taken into account, such as viewpoint problems, illumination problems, deformation, occlusion, background clutter, and interclass variation.

More than 50% of HAR relies on sensor-based data rather than vision-based, RFID, and Wi-Fi data, according to a recent review [1]. The current research area of HAR is primarily focused on sensor-based recognition for the following reasons: (i) advancements in sensor technology; (ii) decreased costs and power consumption; (iii) minimal invasion of privacy; (iv) wearables with less infrastructure; (v) modern smartphones with embedded sensors such as accelerometers, gyroscopes, motion sensors, proximity sensors, and ambient light sensors, which are more accurate and reliable.

**Figure 1:**
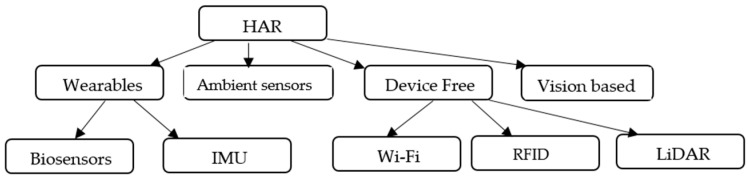
Classification of human activity recognition.

Human activity recognition can be classified as given in Figure 1. Various human activities can be recognized using wearables, ambient sensors, device-free, and video based systems. A number of other sensors, including motion sensors, proximity sensors, and GPS tracking, are also frequently employed to support the HAR system.

Wearables are devices that can be worn on the body, such as smart watches, fitness trackers, or smart clothing. Wearables are popular for HAR due to their ease of use, portability, and continuous monitoring capabilities, and they can even recognize complex activities. Other than inertial measurement sensors, such as an accelerometer, gyroscope, or magnetometer, several biosensors are used for activity recognition, such as the electroencephalogram (EEG), electrocardiogram (ECG), electrocorticogram (ECoG), electrogoniometer, electrooculogram (EOG), and electromyogram (EMG) [2]. Of these, EMG has already been widely used in HAR research because it senses the bioelectrical signals emitted by muscles, and muscle activity is an essential aspect of most human activities. Along with the devices mentioned so far, barometers and various types of microphones, such as piezoelectric (PZT) or airborne, are incorporated for HAR.

Ambient sensors are stationary or fixed sensors placed in an environment where human activities take place. These sensors can include cameras, microphones, infrared motion detectors, and pressure mats. Ambient sensors capture data on movements, sound patterns, or object interactions within the environment. The system can recognize activities without the need for individuals to wear any specific devices in limited coverage areas.

Device-free HAR, also known as deviceless HAR, aims to recognize human activities without requiring individuals to wear any specific sensors or carry any devices. Instead, it leverages existing infrastructure, such as Wi-Fi signals, RFID, or radio frequency signals, to monitor changes in the wireless signals caused by human movements. The variations in these signals can be analyzed through algorithms to identify activities of daily life, or even more complex actions such as dancing or exercising. Device-free HAR can be non-intrusive, but its accuracy might be affected by environmental factors. Channel state information (CSI) refers to the information about the wireless channel characteristics between the transmitter and receiver in a wireless communication system [3]. In device-free HAR, CSI is utilized to monitor changes in the wireless signals caused by the presence and movements of human bodies in the environment.

Vision-based HAR employs cameras or depth sensors (such as Microsoft Kinect) to capture visual data on human activities. Using computer vision and deep learning techniques, the system analyzes the visual information to recognize actions such as hand gestures, facial expressions, or body movements. Vision-based HAR provides rich information but can be computationally intensive and may raise privacy concerns.

Most of the earlier models for HAR are based on shallow neural networks. Different shallow learning algorithms, such as logistic regression, linear regression, decision trees (DT), support vector machines (SVM), hidden Markov models (HMM), and K-nearest neighbors (KNN), have been used over a long time span.

Several studies of HAR have been performed using a hidden Markov model (HMM) and its alternatives. The internal structure of human activity can be better explained by the HMM and its variants, continuous density HMM and hierarchical HMM [4]. A few works have been performed using single-state, multiple-state, and variable-state HMM. Based on the phase and state partitioning of HMM, the type of fall can also be easily determined.

The deep learning algorithms that have gained attention are mainly CNNs [5,6,7] and RNNs [8,9,10,11]. Many other deep learning models, such as deep restricted Boltzmann machine (RBM) [12], deep belief network (DBN) [13], and deep encoder methods [14], have been considered for activity recognition. Discriminative methods such as CNNs, RNNs, and their variants, GRU and LSTM, have gained dominance and the majority of works are conducted using these methods. Many hybrid methods have also been investigated, showing improved performance [15,16,17].

Recently, LiDAR sensors have been widely used for activity recognition [18,19]. LiDAR sensors offer several advantages. Some of the key advantages are 3D sensing, robustness to lighting conditions, occlusion handling, dense and accurate point clouds, a good range and coverage, and multi-person tracking.

RNNs and LSTMs have the drawback of making it difficult to parallelize processing tasks. Moreover, there are some practical drawbacks to CNN-based models, such as a large number of parameters, more learning data, and less expressiveness. Additionally, there is no model that accounts for both long- and short-term dependence.

Transformer-based research studies are also prominent in this area. Transformer models that may extract information about temporal dependencies from sequential data have gained popularity recently. When compared to RNN-series models, the transformer has the advantages of improved computing efficiency and effective long-term dependence information extraction [20,21,22,23].

Gulati et al. [24] 2020 introduced the Conformer, or convolution-augmented transformer. The authors used the model for speech recognition to overcome the limitations of CNNs, transformer and squeeze, and excitation networks; they combined a convolution neural network and self-attention module, inserted between feedforward modules, and they achieved better accuracy with fewer parameters.

Kim Y et al. [25] introduced the Conformer model for HAR. The study showed that the performance of transformer-based HAR can be improved by adding a CNN layer that extracts local features well. The researchers used a time-series data augmentation algorithm, namely the SMOTE algorithm, and they achieved improved performance.

Shang et al. [26] proposed a recurrent Conformer for Wi-Fi channel state information (CSI)-based HAR tasks. The model is specifically suited to learning from a small number of CSI samples and it allows the network to deepen without scale expansion.

Another recent work by Gao et al., 2022 [27] proposed a multi-scale convolution transformer detection method that predicts both the type of activity and its start and end times. The model performs exceptionally well in an indoor setting, with strong micro F1-scores of 92.81% and weak micro F1-scores of 98.37%.

This paper proposes a Conformer-based HAR model that employs attention mechanisms to better capture the temporal dynamics of human movement and improve the recognition accuracy. We modified the basic Conformer method by introducing a sensor attention module at the input to improve the interpretability of sensor data and reduce noisy data. We adopted a framework suitable for practical, wearable, sensor-based HAR, called the HAR-Pipeline, proposed by Liu H. et al. [28]. The model was implemented in Keras and trained on the USCHAD and WISDM datasets, which comprise sensor readings from wearable devices during human activities such as walking, sitting, and jumping. The proposed model consists of convolutional layers, multiple Conformer blocks with self-attention and residual connections, and classification layers. Cross-entropy loss was used to train the model, which was optimized with the Adam optimizer. Experimental results show that the proposed model outperformed existing models such as CNN, LSTM, and GRU. The attention mechanisms in the Conformer blocks help the model capture the temporal dynamics of human movement better, and the residual connections prevent vanishing gradients and improve the convergence. The model was evaluated using two publicly available datasets, WISDM and USCHAD.

The key contributions of this paper are as follows:

- This paper proposes a modified Conformer model that utilizes attention mechanisms to process sparse and irregularly sampled multivariate clinical time-series data;
- The model employs a sensor attention unit that is designed for time-series data from various sensor readings, utilizing the multi-head attention mechanism inherent in transformers;
- The performance of the proposed model is evaluated using two publicly available ADL datasets, USCHAD and WISDM, and the model achieves state-of-the-art prediction results.

The remaining sections of this paper are structured as follows. Section 2 presents a description of the activity recognition chain. In Section 3, Conformers and the proposed approach are described. Section 4 deals with the results and discussion, and the conclusions and future directions constitute Section 5.

## 2. Method

**Figure 2:**
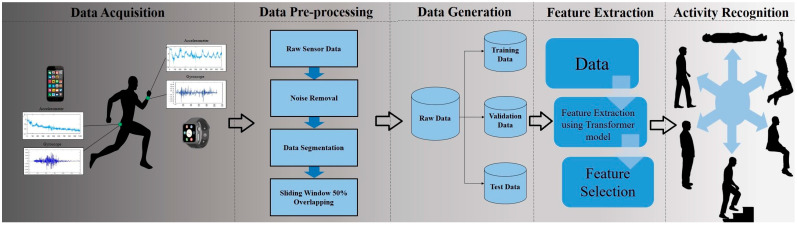
Process for human activity recognition (ARC).

Based on ARC, the many phases of the HAR process are shown in Figure 2. The above steps are implemented in the proposed model. The activity recognition chain (ARC) paradigm, a systematic methodology for the creation and assessment of activity recognition systems, was introduced by Bulling et al. [29]. The ARC is made up of a series of six essential phases that assist researchers and professionals in creating and assessing activity recognition software.

The six different steps are as follows.

(1) Sensor selection: Selection of appropriate sensors and sensor placements to record information about the desired activity. Activities that focus on walking or using one’s hands, such as eating, can be included. Smartphones and wearables can be worn in the pocket, on the wrist, around the waist, etc. From the wearable device’s accelerometer, magnetometer, and gyroscope readings in three directions, x, y and z are retrieved.

(2) Preprocessing of the data: The sensor data should be cleaned and processed to remove noise and artefacts. From the wearable devices, accelerometer and gyroscope readings in three directions, x, y, and z, are retrieved. Once the raw data are acquired, some of the preprocessing techniques, such as data cleaning, sampling, and windowing, are done. In this work, a 50% overlapping sliding window is implemented. The dataset is divided into test, training, and evaluation sets in the ratio of 8:1:1.

(3) Feature extraction: Locate characteristics or patterns in the sensor data that are important in identifying the desired activity. Recent models use automatic feature extraction using deep learning models.

(4) Activity inference: Using various statistical or learning algorithms, determine the activity based on the features that were extracted. The activities are classified and activity inferences are made.

(5) Post-processing: To increase the accuracy of the activity recognition results, further process and polish them. 

(6) Evaluation: Using the proper metrics and benchmark datasets, assess the effectiveness of the activity recognition system. The many phases of activity recognition are depicted in Figure 1.

## 3. Proposed Model

In this paper, we propose a Conformer-based HAR model that integrates attention mechanisms to capture the temporal dynamics of human movement and improve the recognition accuracy.

### 3.1. Conformer

The Conformer convolutional transformer is an advanced neural network architecture that combines the power of convolutional neural networks (CNNs) and transformers. It is designed to address the limitations of traditional convolutional and transformer models in processing sequential data, such as speech and natural language.

Convolutional neural networks excel in capturing local patterns and extracting hierarchical features from input data. They are commonly used in computer vision tasks, where they perform convolution operations on input images to extract relevant visual features. However, CNNs have a limited ability to model long-range dependencies and capture global contexts, which are crucial in understanding sequential data.

On the other hand, transformers have emerged as a powerful architecture for the modeling of sequential data, such as language translation and text generation. Transformers leverage self-attention mechanisms to capture global dependencies and enable the parallel processing of input sequences. However, transformers can be computationally expensive and less effective in capturing local patterns compared to CNNs.

The Conformer architecture combines the strengths of both CNNs and transformers to overcome their respective limitations. It introduces a novel convolutional module called “depth-wise separable convolution”, which performs convolution operations on individual input channels independently. This allows the Conformer to capture local patterns efficiently while maintaining computational scalability.

Additionally, the Conformer employs a modified version of the transformer’s self-attention mechanism called “relative position encoding”. This mechanism takes into account the relative positions of tokens in the input sequence, enabling the model to better understand the contextual relationships between tokens.

The Conformer architecture also incorporates feed forward neural networks and layer normalization, which contribute to the overall model’s stability and performance.

The Conformer convolutional transformer has achieved state-of-the-art results in various sequential data tasks, including automatic speech recognition, natural language understanding, and language translation. Its ability to capture both local and global dependencies makes it a highly effective model for the processing of sequential data in a wide range of applications, such as images, time-series data, etc.

### 3.2. Conformer Block

**Figure 3:**
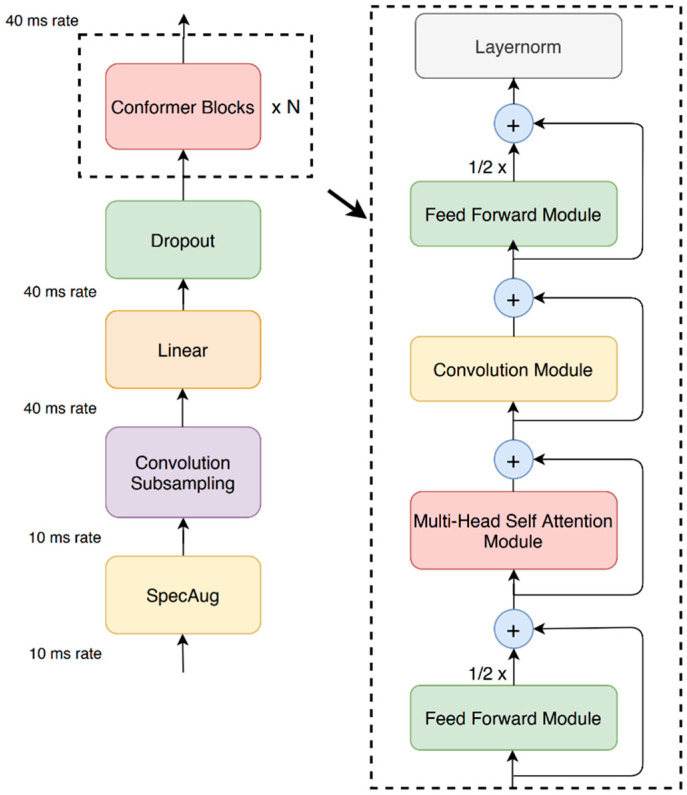
Conformer encoder model architecture. The conformer consists of two feed forward layers that resemble macarons, with half-step residual connections established between the convolution and multi-head self-attention modules [24].

Our proposed Conformer block contains three layers of sequential self-attention with a 1D convolutional layer. Feed forward modules sandwich the multi-head self-attention module and the convolution module, as shown in Figure 3.

This model proposes replacing the original feed forward layer in the transformer block into two half-step feed forward layers, one before the attention layer and one after [30]. The model employs half-step residual weights in our feed forward (*FFN*) modules. The second feed forward module is followed by a final layer, the norm layer. Analytically, for *m_i_* to the Conformer block *i*, the output *n_i_* of the block is

$$\tilde{m_{i}}=m_{i}+\frac{1}{2}FFN\left(m_{i}\right)$$

$$m_{i}^{\prime }=\tilde{m_{i}}+MHSA(\tilde{m_{i}})$$

$$m_{i}^{\prime \prime }=m_{i}^{\prime }+Conv(m_{i}^{\prime })$$

$$n_{i}=LayerNorm(m_{i}^{\prime \prime }+\frac{1}{2}FFN\left(m_{i}^{\prime \prime }\right))$$

where *FFN* refers to the feed forward module, *MHSA* is the multi-head self-attention module, and *Conv* is the convolution module.

### 3.3. Sensor Attention

Sensor attention is a technique used in transformers for activity recognition to help the model to focus on relevant sensor data while ignoring noise and irrelevant data.

However, the raw sensor data collected from multiple sources may contain noise or corrupted data, which makes it difficult for the model to accurately classify the activity.

Attention addresses this issue by allowing the model to selectively attend to specific sensor data based on their relevance to the activity being classified. This is done by computing attention weights for each sensor channel, which are used to weight the input data before they are processed by the transformer layers. The attention weights are typically computed based on a combination of spatial and temporal information. Spatial attention is used to weight each sensor channel based on its relevance to the current activity, while temporal attention is used to weight each time step based on its relevance to the overall activity sequence. By using sensor attention, transformers for activity recognition can improve their accuracy and robustness to noise and irrelevant data in the input sensor streams.

**Figure 4:**
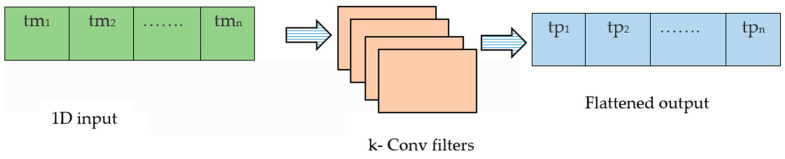
Architecture for sensor attention.

The input is converted into a single channel and *k* convolutional filters are applied with sufficient padding to produce an output with *k* channels. The *k* channels are flattened back to produce a single channel output. The sensor-based SoftMax equation, given below, is used to provide attention weights, which also allows us to plot feature maps [31]. The architecture for the sensor attention block is given in Figure 4

(1) $$s_{k}^{t_{pi}}=\frac{exp({q_{k}}^{\left(t_{mi}\right)})}{\sum_{k}exp({q_{k}}^{\left(t_{mi}\right)})}$$

where *k* represents individual sensors.

**Figure 5:**
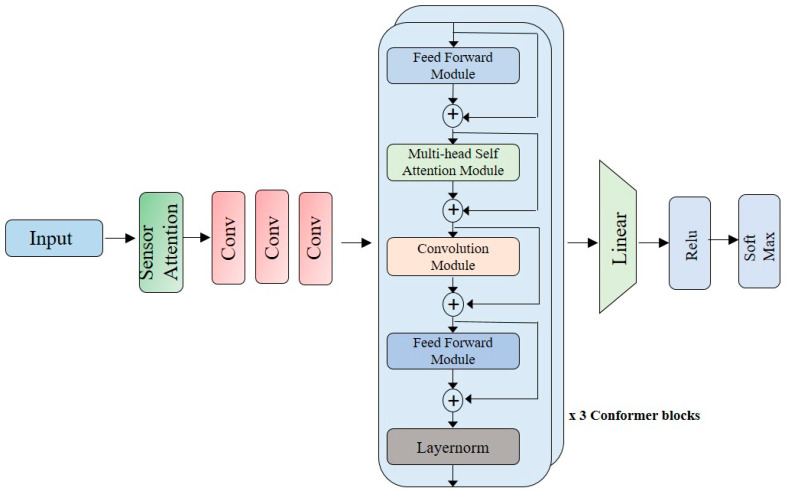
Proposed architecture for the model.

The paper proposes a Conformer-based model for the recognition of human activities using inertial sensors. The model is evaluated on two datasets, USCHAD and WISDM. Input is given to the sensor modality attention block, which is convoluted using one-dimensional convolution modules. Three convolution modules are used. The convoluted block is applied to the Conformer block. Three layers of Conformer blocks are used in the architecture. Finally, flatten, ReLu, and SoftMax layers are used for the classification of different activities. Figure 5 illustrates the architecture of the proposed model.

### 3.4. HAR: Online and Offline

Online mode is preferred when critical analysis is required from real-time data, and this method is resource-intensive. A few works [32,33] have discussed the recognition of human activities in real-time mode. The studies by Liu et al. provide a simple demonstration of real-time HAR using HMM [34], which can be implemented using moderate hardware. In the paper by Ravi et al. [35], a HAR technique based on a deep learning methodology is designed to enable accurate and real-time classification for low-power wearable devices. When the critical analysis of real-time data is necessary, online mode is preferred.

In this work, offline mode is used for the training of the model and recognition of ADL from data collected from sensors, utilizing two datasets: WISDM and USCHAD.

### 3.5. Experimental Setup

The proposed model is shown in Figure 4. It is composed of a sensor attention block and convolutional layers at the input. Three Conformer layers are used for the attention mechanism and finally a classification layer is proposed at the output. The classifier head contains a flatten layer with the ReLu nonlinearity function, and the final output is passed through a SoftMax activation function. The recordings are segmented to 5 s windows with 50% overlap using the sliding window method. The minimum window size that gives good performance for the classifier is a 5 s window and so the best value is given. The dataset is split into three groups, i.e., train, validation, and test sets, with a ratio of 8:1:1, while ensuring that all classes are represented in each section. The weight decay is set to 0.0001, and a batch size of 256 is used during the experiment. The Adam optimizer is used during training. The proposed model utilizes three Conformer blocks, where a fixed attention width of 32 is required for the attention mechanism. The convolutional module has 256 kernels for each layer. During training, the Adam optimizer is used with a learning rate of 0.001.

A learning rate of 0.0001 is initially selected and then it is further reduced using Keras’ ReduceLROnPlateau. The model is trained for 50 epochs. The experiments are performed using Keras in the Google Colab environment, utilizing a GPU with 15 GB RAM. The advantage of the GPU processor present in Colab is utilized and the training period is completed in a reduced time span.

### 3.6. Datasets

Two publicly available datasets using inertial measurement units are used for this study, USCHAD [36] and WISDM [37]. The USCHAD dataset contains data collected from sensors that were attached to participants’ wrists, ankles, and waist. The data include measurements from both accelerometers and gyroscopes and were taken at a frequency of 100 Hz. The data are divided into 12 activity classes, such as walking, jogging, jumping, climbing stairs, sitting, standing, lying down, and other activities. The Wireless Sensor Data Mining (WISDM) dataset is a publicly available dataset for human activity recognition that consists of accelerometer and gyroscope sensor data collected using smart phones and smart watches. The participants performed 18 different activities, each lasting three minutes, while wearing the smart watch or carrying the smartphone. A 20-Hz sampling frequency was used for the accelerometer. The data were collected at a rate of 20 samples per second using a sliding window approach. The dataset includes 18 distinct human activities, which are grouped into three sets: ambulation-related activities, hand-oriented activities excluding eating, and hand-oriented activities while eating. The target variable used for activity recognition is the activity code, and the predictor variables used are the sensor readings in the x, y, and z dimensions of both the gyroscope and accelerometer. The purpose of grouping the activities into sets is to enable the effective analysis of the data.

### 3.7. Evaluation Parameters

The process of evaluating a model is crucial in determining its performance and identifying any potential issues. While accuracy is a commonly used evaluation metric for classification, other important metrics, such as recall, precision, and F1-score, should also be considered. In multiclass classification problems, the confusion matrix is used to calculate these metrics, including recall (sensitivity), accuracy, specificity, precision, and the area under the ROC curve (AUC-ROC). The F1-score is also commonly used to achieve an optimal balance between precision and recall, especially in cases of an uneven class distribution. In this research work, the confusion matrix is plotted and we calculate the precision, recall, and F1-score as evaluation parameters.

The mathematical equations for the aforementioned metrics are as follows:

$$\mathrm{Accuracy}=\frac{TP+TN}{TP+FN+TN+FP}$$

$$\mathrm{Recall}=\frac{TP}{TP+FN}$$

$$\mathrm{Precision}=\frac{TP}{TP+FP}$$

$$F-\mathrm{measure}=\frac{2*Precision*Recall}{Precision+Recall}$$

where *TP* stands for True Positive, *TN* for True Negative, *FN* for False Negative and *FP* for False Positive.

## 4. Results and Discussion

**Figure 6:**
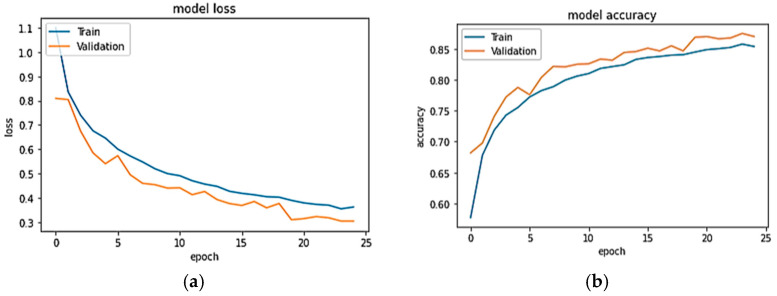
(**a**,**b**). Accuracy and loss plot for USCHAD.

**Figure 7:**
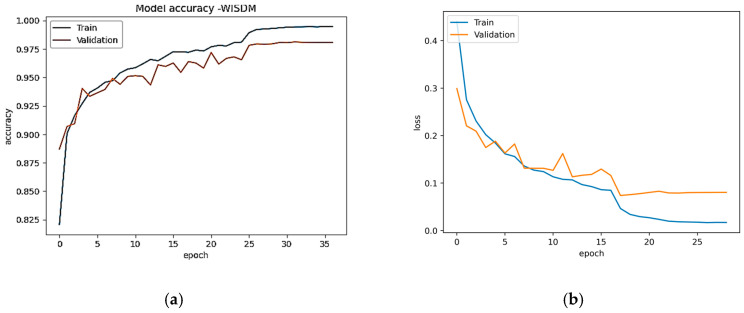
(**a**) Accuracy plot—WISDM. (**b**) Loss plot—WISDM.

The recall, accuracy, and F1-score evaluation results for the proposed Conformer model are shown. The outcomes demonstrate that the Conformer model performs better than other deep learning models, the transformer model, and hybrid models such as CNN-GRU and CNN-BiLSTM. Figure 6a,b show the model accuracy and loss plot of USCHAD, and Figure 7a,b illustrate the accuracy and loss plot of the WISDM dataset obtained in all the epochs during both training and evaluation. From the learning curve, it is clear that the model is under the appropriate conditions, and the training performance of the neural network is good. For the validation and test data, the WISDM smart watch obtained accuracy of 98.06% and 98.19% respectively. Concerning the WISDM dataset, 43,854 samples were utilized for training, 14,618 samples for evaluation, and the same number for testing.

With a total trainable number of parameters of 1,190,918, the proposed model takes only 147 s of epoch time, with a comparable value in the WISDM dataset, achieving the maximum accuracy of 98.19%.

**Figure 8:**
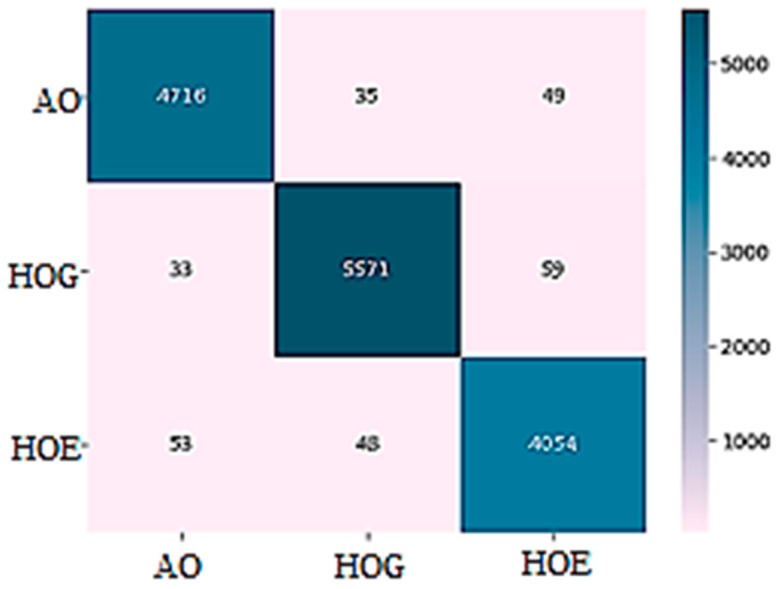
Confusion matrix of WISDM.

**Figure 9:**
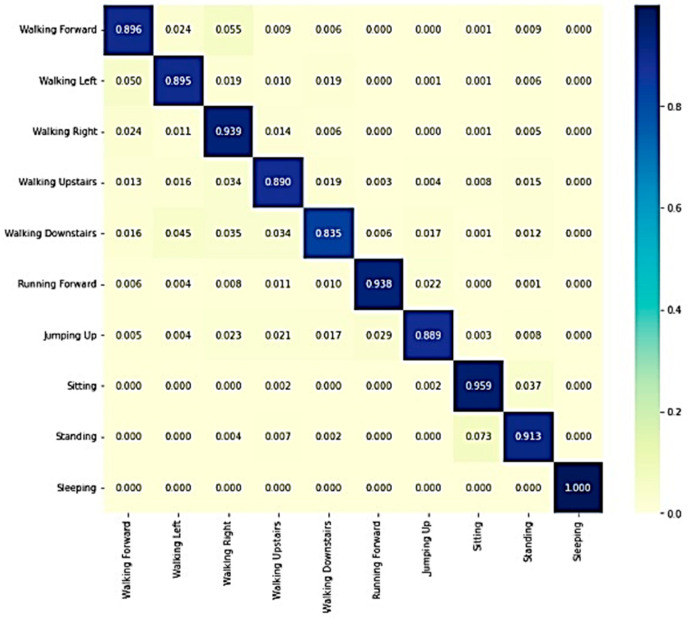
Confusion matrix of USCHAD.

Figure 8 and Figure 9 show the confusion matrixes of the WISDM and USCHAD datasets, which demonstrate that the diagonal elements have a higher proportion than the others, showing that the model is successful in identifying human activities. In conclusion, the proposed transformer model outperforms all other models, and the confusion matrix further supports its efficacy in identifying human activity.

The evaluation parameters for the USCHAD dataset are given in Table 1. The precision, recall, and F1-score are given for both the validation and test data. The table also shows the macro average and weighted average of all the evaluation parameters. The evaluation parameters for the WISDM dataset are given in Table 2.

The performance of the proposed Conformer model is evaluated and compared with various hybrid models [38] in Table 3, using two datasets, USCHAD and Smartphone WISDM. The evaluation shows that the proposed model achieves an accuracy improvement of approximately 96% and 98.1% on these two datasets, respectively. In the studies of [38], it was proven that bidirectional neural networks work better than their unidirectional counterparts.

Different hybrid neural network models are tested, and the outcomes are compared with the suggested Conformer model, which makes use of the attention mechanism for time-series data categorization, to assess the efficacy of the Conformer model.

Table 4 shows the comparison of the proposed approach with related previous works. The performance of the proposed approach is 98.2% for the WISDM dataset. This is in line with the attention-based multi-head model’s 98.2% accuracy, which achieved the best performance in earlier experiments.

## 5. Conclusions

The purpose of this research was to propose a modified Conformer model for the classification of complex human activities. Two different datasets, USCHAD and WISDM, were used in this study. Separate datasets related to smart watches and smart phones were separated from the WISDM original dataset. The manuscript proposes a modified transformer model that is presented as a promising alternative to existing deep learning models such as recurrent neural networks or convolutional neural networks. The model is shown to offer a better representation of time-series data and can scale to more than one million parameters, depending on the number of classes. It is also suitable for low-power real-time applications and does not require pre-transformation of the data, allowing them to be fed directly to the neural network after normalization. Furthermore, the transformer model can be parallelized for execution on a GPU.

The study found that the Conformer model was effective in categorizing human activities using the WISDM and USCHAD datasets, which contain a diverse range of daily life activities. The adapted Conformer model demonstrated high levels of accuracy and precision, indicating its potential as a cutting-edge technology for human activity recognition.

In future studies, it would be valuable to evaluate the adapted Conformer model on a larger dataset with a wider range of sensor data, to assess its performance and generalizability. Additionally, it would be beneficial to explore various transformer models from Hugging Face using different datasets. The model’s pre-training efficiency could also be utilized for real-time applications using IoT wearables, potentially leading to innovative applications such as direct support for humans.

## Figures and Tables

**Table 1:** Evaluation parameters for USCHAD.

	Precision	Recall	F1-Score
Walking Forward	0.92	0.94	0.93
Walking Left	0.90	0.97	0.93
Walking Right	0.96	0.92	0.94
Walking Upstairs	0.98	0.93	0.95
Walking Downstairs	0.96	0.95	0.96
Running Forward	0.98	0.97	0.97
Jumping Up	0.94	0.97	0.95
Sitting	0.99	0.99	0.99
Standing	0.92	0.96	0.94
Sleeping	1.00	1.00	1.00
Micro avg	0.96	0.96	0.96
Macro avg	0.95	0.96	0.96
Weighted avg	0.96	0.96	0.96

**Table 2:** Evaluation parameters for WISDM.

Smart Watch Results with Conformer for Validation Set			
	Precision	Recall	F1-Score
Ambulation-Related	0.9815	0.9803	0.9809
Hand-Oriented Eating	0.9763	0.9724	0.9744
Hand-Oriented Eating	0.9828	0.9866	0.9847
Accuracy			0.9806
Macro avg	0.9802	0.9798	0.9800
Weighted avg	0.9806	0.9806	0.9806
**Smart Watch Results with Conformer for Test Set**			
	**Precision**	**Recall**	**F1-Score**
Ambulation-Related	0.9819	0.9800	0.9810
Hand-Oriented Eating	0.9788	0.9758	0.9733
Hand-Oriented Eating	0.9841	0.9879	0.9860
Accuracy			0.9819
Macro avg	0.9816	0.9813	0.9814
Weighted avg	0.9819	0.9819	0.9819

**Table 3:** Comparison of results with two datasets, USCHAD and WISDM [38].

USCHAD		WISDM	
Method	Accuracy	Method	Accuracy
CNN-LSTM	90.6	CNN-LSTM	88.7
CNN-BiLSTM	91.8	CNN-BiLSTM	89.2
CNN-GRU	89.1	CNN-GRU	89.6
CNN-BiGRU	90.2	CNN-BiGRU	90.2
Transformer model	91.2	Transformer model	90.8
Proposed method	96.0	Proposed method	98.2

**Table 4:** Comparison of previous algorithms and proposed model.

Algorithm	WISDM Accuracy %
Proposed	98.2
Fusion-Mdk-ResNet [39]	96.8
Deep CNN-RF [40]	97.7
Attention-based multi-head [41]	98.2

## Data Availability

Sensor-based HAR datasets including WISDM [34] and USCHAD [35], which are openly accessible for use in research, were employed in the studies.
